# TOM70 in Glial Cells as a Potential Target for Treatment of COVID-19

**DOI:** 10.3389/fncel.2021.811376

**Published:** 2021-12-24

**Authors:** Yorran Hardman Araújo Montenegro, Geancarlo Zanatta, André Quincozes-Santos, Guilhian Leipnitz

**Affiliations:** ^1^Programa de Pós-Graduação em Neurociências, Universidade Federal do Rio Grande do Sul, Porto Alegre, Brazil; ^2^Programa de Pós-Graduação em Bioquímica, Universidade Federal do Ceará, Fortaleza, Brazil; ^3^Departamento de Física, Universidade Federal do Ceará, Fortaleza, Brazil; ^4^Programa de Pós-Graduação em Ciências Biológicas: Bioquímica, Universidade Federal do Rio Grande do Sul, Porto Alegre, Brazil

**Keywords:** neurological symptoms, glial cells, mitochondria, TOM70, SARS-CoV-2, COVID-19

## Introduction

The coronavirus disease 2019 (COVID-19) pandemic caused by the severe acute respiratory syndrome coronavirus 2 (SARS-CoV-2) brought a new health challenge to the world. One of the main clinical concerns associated with this pathology is the heterogeneity of symptoms. Although the respiratory tract is the main target, central nervous system (CNS) involvement has raised special interest since neurological symptoms have been reported in over 30% of hospitalized patients and ~85% of patients with acute respiratory distress syndrome (Iadecola et al., 2020; Balcom et al., 2021). Acute neurological signs include anosmia, ageusia, headache, altered mental status, seizures, and stroke (Iadecola et al., 2020; Balcom et al., 2021). Interestingly, headache, fatigue, dysgeusia, and anosmia are common in both mild and severe cases (Kanberg et al., 2021). However, it is yet to be established whether altered mental health is a result from an encephalopathy caused by a systemic inflammatory condition or an encephalitis caused by SARS-CoV-2 neuroinvasion (Iadecola et al., 2020; Balcom et al., 2021). Noteworthy, viral infections may cause neurologic impairment through direct infection of different cells, including neurons, glia or endothelial cells, resulting in acute cell death (Iadecola et al., 2020).

Despite the evidence reporting various neurological manifestations in COVID-19, it is still uncertain whether SARS-CoV-2 is neurotropic or elicits its effects through the excessive immune response since cytokines can directly pass the blood-brain barrier (BBB) (Iadecola et al., 2020; Balcom et al., 2021). Consistent with the neurotropism hypothesis, previous findings demonstrated that angiotensin-converting enzyme 2 (ACE2) receptor, the protein by which SARS-CoV-2 infects cells, is expressed at relatively high levels in neurons and glial cells of several brain areas as well as in vascular wall cells (Iadecola et al., 2020; Chen et al., 2021). It should be also noted that SARS-CoV-2 may use alternative docking receptors, such as neuropillin-1 and basigin, that are found at high levels in the brain (Balcom et al., 2021).

The observations above also highlight that BBB vascular endothelium is one of the main candidates to mediate SARS-CoV-2 neuroinvasion (Balcom et al., 2021; Jha et al., 2021). The BBB is a complex formed by specialized cerebral microvascular endothelial cells, perivascular cells (pericytes) and astrocyte end-feet responsible for separating the peripheral blood supply and the CNS and regulating the bidirectional flow of molecules between these compartments (Liebner et al., 2018). Of note, interneurons and perivascular microglia also make contacts with BBB forming the so-called neurovascular unit (Liebner et al., 2018). It has been proposed that the neuroinvasion of SARS-CoV-2 through the BBB may occur via interaction of the viral spike protein with ACE2 expressed in the capillary endothelium, subsequently infecting CNS cells, including glia (Jha et al., 2021). Consistent with this, the receptors involved in SARS-CoV-2 infection including ACE2 were seen to be expressed in the cells of the neurovascular unit, especially in astrocytes and microglial cells (Torices et al., 2021). Recent studies also showed that human pericyte-like cells from brain organoids and endothelial cells from mouse cerebral microvessels can be infected by SARS-CoV-2 (Wang et al., 2021; Wenzel et al., 2021). Interestingly, it was verified that ACE2 was predominantly localized in the pericytes from the mouse microvessels (Wenzel et al., 2021). Further studies showed that SARS-CoV-2 is able to directly infect neural stem cell-derived astrocytes (Crunfli et al., 2020) and hamster astrocytes (de Oliveira et al., 2021), and that SARS-CoV-2 spike protein was present in different cells from brain of patients, the majority of these cells being astrocytes (Crunfli et al., 2020).

Alternatively, pro-inflammatory cytokines can disrupt BBB and enter the CNS, inducing astrocyte and microglia activation, which can further disrupt BBB and facilitate SARS-CoV-2 neuroinvasion (Iadecola et al., 2020; Balcom et al., 2021). Consistent with this, the integrity of the BBB is compromised in multiple conditions associated with mortality in COVID-19, including hypertension, diabetes, smoking, and stroke (Yang and Rosenberg, 2011; Iadecola et al., 2020; Balcom et al., 2021). Furthermore, areas of increased vascular permeability or lack of BBB, such as the pituitary and median eminence of the hypothalamus, are rich in ACE2 (Doobay et al., 2007).

## Glial Cells in the Pathophysiology of COVID-19

Although the pathophysiological mechanisms underlying the neurological signs in COVID-19 are still unknown, mounting evidence suggests that glial cells might have a crucial role. Specifically, astrocytic injury was observed in the acute phase of COVID-19, as shown by high plasma levels of glial fibrillary acidic protein (GFAP), with more pronounced findings in hospitalized patients (Kanberg et al., 2020). A recent study further verified that, while astrocytic injury or activation occurs early in the acute phase of COVID-19, neuronal damage continued to progress for a longer period (Kanberg et al., 2021). It was also found that, in the presence of pericyte-like cells in cortical organoids, astrocytes showed vulnerability to SARS-CoV-2 infection, undergoing apoptosis or activating inflammatory signaling (Wang et al., 2021). Importantly, it was demonstrated that SARS-CoV-2-infected neural stem cell-derived astrocytes had alterations in bioenergetics and changes in key proteins and metabolites that are crucial for neurotransmitter synthesis, which secondarily impacted neurons (Crunfli et al., 2020). Changes in energy metabolism, as well as in carbon metabolism were also revealed in hamster astrocytes with SARS-CoV-2 (de Oliveira et al., 2021).

In addition, autopsy findings also revealed astroglial and microglial activation throughout the brain, accompanied by infiltration of cytotoxic T cells (Matschke et al., 2020; Pröbstel and Schirmer, 2021), indicating that cytokine storm might have a crucial role in the neuroinflammatory process. Moreover, inflammatory CNS syndromes that may be caused by direct infection of glial cells, including encephalitis, acute disseminated encephalomyelitis and myelitis, were further observed in COVID-19 patients (Balcom et al., 2021). Thus, it is reasonable to hypothesize that glial cells might be an early target of SARS-CoV-2 infection.

## Is TOM70 Blockage in Glia Implicated in the Neurological Manifestations?

TOM70 is a multifunctional protein of the translocase of the outer membrane (TOM) complex that recognizes and cooperates with the molecular chaperone heat shock protein (Hsp) 90 to transfer preproteins from cytosol to mitochondria (Neupert and Herrmann, 2007). In addition to this important function, TOM70 also plays an important role in activating innate immune response to viral infections. It acts as a key adapter that relays antiviral signaling from the mitochondrial antiviral signaling protein (MAVS) to TANK-binding kinase 1 (TBK1)/interferon regulatory factor 3 (IRF3) (Liu et al., 2010). The importance of this mechanism is not only associated with the inflammatory process, but may be also involved in the pathology of COVID-19 itself. Interestingly, other viruses have been shown to modulate TOM70, such as the hepatitis C virus (Kasama et al., 2012). Regarding SARS-CoV-2, it was observed that the Alpha variant hosts genomic mutations responsible for increasing up to 16-fold the expression of an open reading frame encoding a small accessory protein called Orf9b (Parker et al., 2021), which binds to TOM70 (Gordon et al., 2020) and leads to the suppression of the innate immune response (Thorne et al., 2021). Structurally, Orf9b undergoes modifications from dimeric state in cytoplasm to monomeric state interacting with TOM70 (Gao et al., 2021). It is speculated that Orf9b initially interacts with the site for the recruitment of chaperone-associate preproteins, at the N-terminal TRP domain of TOM70, where it induces the transition of TOM70 from “close” to the “open” state (Gao et al., 2021). Next, Orf9b performs a translocation to the binding site within TOM70 C-terminal, where a serine in the position 53 of Orf9b interacts with a glutamate at position 477 of TOM70 to lock the receptor in the “open” state (Brandherm et al., 2021). It seems that, although the increasing Orf9b expression is unlikely to impact transmission rate, it could impair the innate immune response by blocking TOM70.

Although expression levels of TOM70 in astrocytes are not known, interferon (IFN) responses have been widely reported to be produced in these cells (Owens et al., 2014). In viral encephalitis, for example, IFNI acts directly on the microglial activation profile via astrocytic signaling (Chhatbar et al., 2018). Another example of this communication is related to the Zika virus, which, through signaling processes, prevents the activation of the glial inflammatory process via the STAT pathway (Kumar et al., 2016), initiated by the influence of IFNI released by mitochondrial processes associated with glia (Owens et al., 2014). In fact, in both examples, there is an intrinsic relationship between the inflammatory process, glia and TOM70. Therefore, it is conceivable that immune evasion by targeting glial TOM70 may have a crucial role in the onset and development of the neurological dysfunction verified in many COVID-19 patients.

## Concluding Remarks

**Figure 1:**
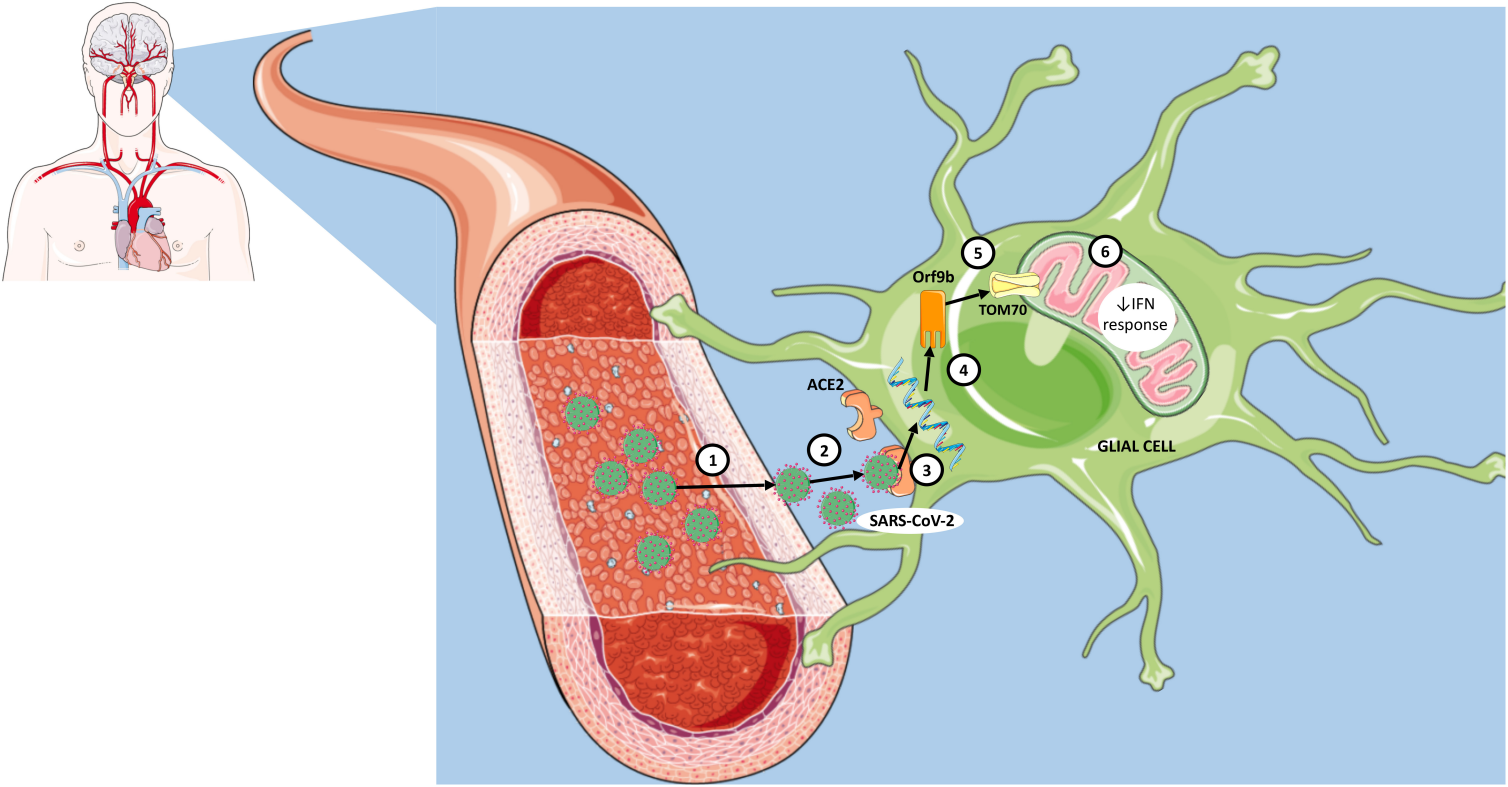
Proposed steps for interaction of SARS-CoV-2 with mitochondrial TOM70 in glia and consequent viral immune evasion. (1) Infection of blood-brain barrier endothelial cells by SARS-CoV-2. (2) Entry of SARS-CoV-2 into the structures of the central nervous system. (3) Recognition of angiotensin-converting enzyme 2 (ACE2) receptors in neural cells, including glia. (4) Insertion of genetic material into cell cytoplasm. (5) Translation of Orf9b and subsequent interaction with TOM70 (mitochondrial membrane protein). (6) Impaired mitochondrial innate immune response via interferon (IFN) signaling.

Since mounting evidence shows that COVID-19 causes neurological dysfunction and may lead to long-term neurological sequelae, there is an urgent need to find novel targets that offer the perspective for the development of effective therapeutic strategies. Here, we want to shed light on the potential role of TOM70 protein, especially from glial cells, in the neuropathology of COVID-19 (Figure 1). Although the neurotropism of SARS-CoV-2 is still under debate, SARS-CoV-2 viral genomes were detected in the brain and cerebrospinal fluid (CSF) of patients, supporting the hypothesis that SARS-CoV-2 is neuroinvasive. Nevertheless, even if SARS-CoV-2 is not neurotropic, we cannot rule out that systemic pro-inflammatory cytokines may also impair TOM70-mediated immune response in glial cells. Finally, it is expected that the elucidation of the consequences of the interaction of SARS-CoV-2 Orf9b with mitochondrial TOM70 in glial cells may provide important insights on the pathomechanisms of COVID-19, as well as potential treatments for this disease.

## Author Contributions

YM created the figure. All authors wrote and reviewed the manuscript.

## Funding

YM was funded by CAPES. GZ was funded by CNPq (437373/2018-5). GL and AQ-S were funded by CAPES (014330/2020-11) and FAPERGS (21/2551-0000067-8).

## Conflict of Interest

The authors declare that the research was conducted in the absence of any commercial or financial relationships that could be construed as a potential conflict of interest.

## Publisher's Note

All claims expressed in this article are solely those of the authors and do not necessarily represent those of their affiliated organizations, or those of the publisher, the editors and the reviewers. Any product that may be evaluated in this article, or claim that may be made by its manufacturer, is not guaranteed or endorsed by the publisher.

## References

[B1] Balcom E. F. Nath A. Power C. (2021). Acute and chronic neurological disorders in COVID-19: potential mechanisms of disease. Brain. awab302. 10.1093/brain/awab302 34398188 PMC8719840

[B2] Brandherm L. Kobaš A. M. Klöhn M. Brüggemann Y. Pfaender S. Rassow J.. (2021). Phosphorylation of SARS-CoV-2 Orf9b regulates its targeting to two binding sites in TOM70 and recruitment of Hsp90. Int. J. Mol. Sci. 22:9233. 10.3390/ijms22179233 34502139 PMC8430508

[B3] Chen R. Wang K. Yu J. Howard D. French L. Chen Z.. (2021). The spatial and cell-type distribution of SARS-CoV-2 receptor ACE2 in the human and mouse brains. Front. Neurol. 11:573095. 10.3389/fneur.2020.573095 33551947 PMC7855591

[B4] Chhatbar C. Detje C. N. Grabski E. Borst K. Spanier J. Ghita L.. (2018). Type I interferon receptor signaling of neurons and astrocytes regulates microglia activation during viral encephalitis. Cell Rep. 25, 118.e4–129.e4. 10.1016/j.celrep.2018.09.003 30282022 PMC7103936

[B5] Crunfli F. Carregari V. C. Veras F. P. Vendramini P. H. Valença A. G. F. Antunes A. S. L. M.. (2020). SARS-CoV-2 infects brain astrocytes of COVID-19 patients and impairs neuronal viability. medRxiv. [preprint]. 10.1101/2020.10.09.20207464

[B6] de Oliveira L. G. de Souza Angelo Y. Yamamoto P. Carregari V. C. Crunfi F. Reis-de-Oliveira G.. (2021). SARS-CoV-2 infection impacts carbon metabolism and depends on glutamine for replication in syrian hamster astrocytes. bioRxiv. [preprint]. 10.1101/2021.10.23.465567 PMC9350388 35880385

[B7] Doobay M. F. Talman L. S. Obr T. D. Tian X. Davisson R. L. Lazartigues E. (2007). Differential expression of neuronal ACE2 in transgenic mice with overexpression of the brain renin-angiotensin system. Am. J. Physiol. Regul. Integr. Comp. Physiol. 292, R373–R381. 10.1152/ajpregu.00292.2006 16946085 PMC1761128

[B8] Gao X. Zhu K. Qin B. Olieric V. Wang M. Cui S. (2021). Crystal structure of SARS-CoV-2 Orf9b in complex with human TOM70 suggests unusual virus-host interactions. Nat. Commun. 12:2843. 10.1038/s41467-021-23118-8 33990585 PMC8121815

[B9] Gordon D. E. Hiatt J. Bouhaddou M. Rezelj V. V. Ulferts S. Braberg H.. (2020). Comparative host-coronavirus protein interaction networks reveal pan-viral disease mechanisms. Science 370:eabe9403. 10.1126/science.abe9403 33060197 PMC7808408

[B10] Iadecola C. Anrather J. Kamel H. (2020). Effects of COVID-19 on the nervous system. Cell 183, 16.e1–27.e1. 10.1016/j.cell.2020.08.028 32882182 PMC7437501

[B11] Jha N. K. Ojha S. Jha S. K. Dureja H. Singh S. K. Shukla S. D.. (2021). Evidence of coronavirus (CoV) pathogenesis and emerging pathogen SARS-CoV-2 in the nervous system: a review on neurological impairments and manifestations. J. Mol. Neurosci. 71, 2192–2209. 10.1007/s12031-020-01767-6 33464535 PMC7814864

[B12] Kanberg N. Ashton N. J. Andersson L. M. Yilmaz A. Lindh M. Nilsson S.. (2020). Neurochemical evidence of astrocytic and neuronal injury commonly found in COVID-19. Neurology 95, e1754–e1759. 10.1212/WNL.0000000000010111 32546655

[B13] Kanberg N. Simrén J. Edén A. Andersson L. M. Nilsson S. Ashton N. J.. (2021). Neurochemical signs of astrocytic and neuronal injury in acute COVID-19 normalizes during long-term follow-up. EBioMedicine 70:103512. 10.1016/j.ebiom.2021.103512 34333238 PMC8320425

[B14] Kasama Y. Saito M. Takano T. Nishimura T. Satoh M. Wang Z.. (2012). Translocase of outer mitochondrial membrane 70 induces interferon response and is impaired by hepatitis C virus NS3. Virus Res. 163, 405–409. 10.1016/j.virusres.2011.10.009 22032846

[B15] Kumar A. Hou S. Airo A. M. Limonta D. Mancinelli V. Branton W.. (2016). Zika virus inhibits type-I interferon production and downstream signaling. EMBO Rep. 17, 1766–1775. 10.15252/embr.201642627 27797853 PMC5283583

[B16] Liebner S. Dijkhuizen R. M. Reiss Y. Plate K. H. Agalliu D. Constantin G. (2018). Functional morphology of the blood-brain barrier in health and disease. Acta Neuropathol. 135, 311–336. 10.1007/s00401-018-1815-1 29411111 PMC6781630

[B17] Liu X. Y. Wei B. Shi H. X. Shan Y. F. Wang C. (2010). TOM70 mediates activation of interferon regulatory factor 3 on mitochondria. Cell Res. 20, 994–1011. 10.1038/cr.2010.103 20628368

[B18] Matschke J. Lütgehetmann M. Hagel C. Sperhake J. P. Schröder A. S. Edler C.. (2020). Neuropathology of patients with COVID-19 in Germany: a post-mortem case series. Lancet Neurol. 19, 919–929. 10.1016/S1474-4422(20)30308-2 33031735 PMC7535629

[B19] Neupert W. Herrmann J. M. (2007). Translocation of proteins into mitochondria. Ann. Rev. Biochem. 76, 723–749. 10.1146/annurev.biochem.76.052705.163409 17263664

[B20] Owens T. Khorooshi R. Wlodarczyk A. Asgari N. (2014). Interferons in the central nervous system: a few instruments play many tunes. Glia 62, 339–355. 10.1002/glia.22608 24588027

[B21] Parker M. D. Lindsey B. B. Shah D. R. Hsu S. Keeley A. J. Partridge D. G.. (2021). Altered subgenomic RNA expression in SARS-CoV-2 B.1.1.7 infections. bioRxiv. [preprint]. 10.1101/2021.03.02.433156

[B22] Pröbstel A. Schirmer L. (2021). SARS-CoV-2-specific neuropathology: fact or fiction? Trends Neurosci. 44, 933–935. 10.1016/j.tins.2021.10.006 34716032 PMC8519811

[B23] Thorne L. Bouhaddou M. Reuschl A. K. Zuliani-Alvarez L. Polacco B. Pelin A.. (2021). Evolution of enhanced innate immune evasion by the SARS-CoV-2 B.1.1.7 UK variant. bioRxiv. [preprint]. 10.1101/2021.06.06.446826 34127972 PMC8202424

[B24] Torices S. Cabrera R. Stangis M. Naranjo O. Fattakhov N. Teglas T.. (2021). Expression of SARS-CoV-2-related receptors in cells of the neurovascular unit: implications for HIV-1 infection. J. Neuroinflammation 18:167. 10.1186/s12974-021-02210-2 34325716 PMC8319595

[B25] Wang L. Sievert D. Clark A. E. Lee S. Federman H. Gastfriend B. D.. (2021). A human three-dimensional neural-perivascular 'assembloid' promotes astrocytic development and enables modeling of SARS-CoV-2 neuropathology. Nat. Med. 27, 1600–1606. 10.1038/s41591-021-01443-1 34244682 PMC8601037

[B26] Wenzel J. Lampe J. Müller-Fielitz H. Schuster R. Zille M. Müller K.. (2021). The SARS-CoV-2 main protease M pro causes microvascular brain pathology by cleaving NEMO in brain endothelial cells. Nat. Neurosci. 24, 1522–1533. 10.1038/s41593-021-00926-1 34675436 PMC8553622

[B27] Yang Y. Rosenberg G. A. (2011). Blood-brain barrier breakdown in acute and chronic cerebrovascular disease. Stroke 42, 3323–3328. 10.1161/STROKEAHA.110.608257 21940972 PMC3584169

